# MicroRNA expression profile of urinary exosomes in Type IV lupus nephritis complicated by cellular crescent

**DOI:** 10.1186/s40709-018-0088-0

**Published:** 2018-10-04

**Authors:** Yi Li, Xiaosong Xu, Xiaopeng Tang, Xiuwu Bian, Bingbing Shen, Hongwen Zhao, Shiyuan Luo, Zhiwen Chen, Keqin Zhang

**Affiliations:** 1 Department of Nephrology, The First Hospital Affiliated To Army Medical University, No. 29 Gaotanyan Street, Sha Ping Ba District, Chongqing, 400038 China; 2 Department of Pathology, The First Hospital Affiliated To Army Medical University, No. 29 Gaotanyan Street, Sha Ping Ba District, Chongqing, 400038 China; 3 Department of Urology, The First Hospital Affiliated To Army Medical University, No. 29 Gaotanyan Street, Sha Ping Ba District, Chongqing, 400038 China

**Keywords:** Type IV lupus nephritis, Urinary exosome, miRNAs, High-throughput sequencing

## Abstract

**Background:**

Type IV lupus nephritis (LNIV) is a severe disease characterized by diffuse proliferative lesions, and its prognosis is worse with cellular crescent (LNIV-CC) involvement. Urinary exosomes have been shown to reflect the degree of kidney injury. This study was aimed to identify non-invasive diagnostic markers for LNIV-CC. We analysed the expression profile of microRNAs (miRNAs) isolated from urinary exosomes in patients with LNIV-CC and LNIV, and healthy individuals using high-throughput sequencing.

**Results:**

A total of 66 differentially expressed miRNAs were identified, which were significantly enriched in 15 signalling pathways. Bioinformatic analysis revealed a co-expression network of miRNAs, predicted transcription factors and target mRNAs. Expression of three miRNAs including miR-3135b, miR-654-5p, and miR-146a-5p were further analysed and validated by reverse transcription-quantitative polymerase chain reaction. ROC analysis suggested these as candidate biomarkers for LNIV-CC.

**Conclusions:**

LNIV-CC has a unique miRNA expression profile of urinary exosome and complex regulatory network. miR-3135b, miR-654-5p and miR-146a-5p in urinary exosomes could be used as novel non-invasive diagnostic markers for LNIV-CC.

## Background

Type IV lupus nephritis (LNIV) is a severe disease characterized by diffuse proliferative lesions. The treatment regimens of LNIV are determined based on pathological features in accordance to the guidelines of “the Kidney Disease: Improving Global Outcomes” (KDIGO) [1]. Recently, it was demonstrated that cellular crescent (CC) in renal biopsy is closely associated with rapid renal failure [2, 3]. Therefore, the 2017 Oxford Classification of IgAN added MEST-C scoring for cellular/fibrous crescents to assess the significant impact of crescents on kidney diseases [4]. However, invasive biopsy cannot be performed frequently in patients with LNIV. Therefore, screening for reliable biomarkers is urgently needed for non-invasive diagnosis and long-term monitoring of LNIV.

Exosomes are small vesicles (diameter of 30–120 nm) containing microRNAs (miRNAs), mRNAs, and proteins [5, 6]. They are found in almost all biological fluids including urine [5, 7]. Exosomes play an important role in the homeostasis and progression of kidney disease, known to interact with RNA-binding proteins or package them for transport [8, 9]. Although some urinary miRNAs are derived from circulation, the majority of miRNAs are derived from nephron exosomes [10, 11]. Since exosomes can be excreted in all cells of the nephrons, analysis of exosomes can accurately reflect renal dysfunction and structural damage [5, 7]. Therefore, miRNAs in urinary exosomes are potential biomarkers for diagnosis and prognosis [10, 11]. Recent studies indicated distinct miRNA expression profiles of urinary exosomes in patients with focal segmental glomerulosclerosis, and Type I and Type II diabetic nephropathy [12, 13]. Moreover, the miR-29c expression level in urinary exosomes was found to predict early fibrosis in lupus nephritis [14].

In the present study, we investigated the expression profiles of miRNAs isolated from urinary exosomes in patients with LNIV-CC and LNIV (without CC), we validated three selected differentially expressed miRNAs by reverse transcription-quantitative polymerase chain reaction (RT-qPCR), and performed bioinformatic analysis on the differentially expressed miRNAs and receiver operating characteristic (ROC) analysis for the validated miRNAs.

## Methods

### Subjects

A total of 44 patients were enrolled at the Department of Nephrology, the First Hospital Affiliated to Army Medical University from February 2017 to January 2018, including 15 active LNIV, 15 active LNIV-CC and 14 inactive LNIV. A total of 13 healthy volunteers without systemic diseases and normal renal function were also recruited from the health centre of the hospital from February 2017 to January 2018. miRNA sequencing was performed on 5 cases of active LNIV, 5 cases of active LNIV-CC, 4 cases of inactive LNIV, and 3 cases of healthy volunteers. The qRT-PCR validation was performed on additional patients (including 10 active LNIV, 10 active LNIV-CC and 10 inactive LNIV) and healthy volunteers (10 cases). All patients with LNIV were diagnosed according to the KDIGO Clinical Practice Guidelines [1], treated and tested by renal biopsy. Inactive nephritis was defined as a proteinuria level of less than 0.5 g day^−1^ with stable renal function without active urinary sediments. The study was approved by the Ethics Committee of the First Hospital Affiliated to Army Medical University (Southwest Hospital) in accordance with the Helsinki Declaration amended in 2008. All participants signed informed consents.

### Urine processing, urinary exosome isolation, and exosomal RNA extraction

Fresh first-morning urine samples (100 ml) were collected in sterile containers and processed within 1 h after collection. Exosomes were isolated from the urine specimens using differential centrifugation. Briefly, the urine sample was centrifuged at 2500×*g* for 30 min to remove the cells and debris, and then centrifuged at 17,000×*g* for 30 min at 4 °C to remove large membrane vesicles. The resulting supernatant was centrifuged (Optima™ MAX-XP Ultracentrifuge, MLA-50 rotor, Beckman Coulter, USA) at 200,000×*g* for 70 min at 4 °C to pellet the exosomes. The resulting pellets were resuspended in phosphate-buffered saline (PBS) and centrifuged at 200,000×*g* for 70 min at 4 °C. Finally, the putative exosome pellets were resuspended in PBS and stored at − 80 °C.

RNA was extracted from the exosomes using Trizol (Invitrogen, Carlsbad, CA, USA) according to the manufacturer’s recommended protocol. Denatured exosomes were spiked with the normalization control, the synthetic *Caenorhabditis elegans* miRNA cel-miR-39 (RiboBio, Guangzhou, China), lacking the endogenous human urinary miRNA. The RNA concentration was determined using the Qubit™ 3.0 Fluorometer (Life Technologies, USA).

### Structural characteristics of exosome

To verify the characteristics of exosomes, we examined the morphology of exosomes using transmission electron microscopy. A drop (~ 30 μl) of exosomes was resuspended in PBS and placed on parafilm. A formvar/carbon-coated copper grid was positioned on top of each drop for 15 min. Finally, a drop of 2% uranyl acetate was placed on the parafilm and incubated the exosomes for 5 min in the dark. The grids were negatively stained, air-dried, and examined with transmission electron microscopy (JEM-1400/JEM-1400 PLUS, Japan).

### Western blot

The urinary exosomes were lysed in RIPA lysis buffer on ice, and then centrifuged at 12,000×*g* for 15 min. The supernatant was collected and transferred to a new EP tube. An equal amount of total soluble protein was subjected to 8% sodium dodecyl sulphate–polyacrylamide gel electrophoresis (Life Technologies, USA) and transferred to a polyvinylidene fluoride membrane (Millipore, USA). The membranes were incubated with primary antibodies of CD9 and CD81 (CD9 rabbit, dilution 1:1000; CD81 rabbit, dilution 1:1000) and a primary antibody of non-exosomal protein calnexin mouse polyclonal (dilution 1:1000; Abcam, UK). Then, the membranes were incubated with appropriate horseradish peroxidase-conjugated secondary antibodies. Blots were visualized with chemiluminescence reagents (Beyotime, Shanghai, China).

### miRNA sequencing

Total RNA was extracted from exosomes and used for miRNA high-throughput sequencing. Library preparation and miRNA sequencing were performed by Ribobio (Guangzhou, China). Briefly, total RNA samples were fractionated and only small RNAs were used for library preparation. After PCR amplification, the products were sequenced using the Illumina HiSeq 2500 platform (Illumina Inc., San Diego, CA, USA) following the manufacturer’s instruction on running the instrument. Raw sequencing reads were obtained by using related Illumina’s analysis software.

### Transcription factor (TF)–miRNA–mRNA network analysis

Differentially expressed exosomal miRNAs were screened out from the sequencing results. To predict TFs of the identified 66 differentially expressed miRNAs, BioPython [15] of JASPAR Database [16], FIMO [17] of MEME software [18], and Transcription Element Search System from the Computational Biology and Informatics Laboratory at the University of Pennsylvania were used. TargetScan (Release 7.2; http://www.targetscan.org/vert_72/), miRDB (http://mirdb.org/), miRTarBase (Release 7.0; http://mirtarbase.mbc.nctu.edu.tw/php/index.php), and miRWalk (Version 7.0; http://mirwalk.umm.uni-heidelberg.de/) software tools were used to predict the target gene of the identified differentially expressed miRNAs. Cytoscape (Version 3.6.1; http://www.cytoscape.org/) was used to establish miRNA-based regulatory network. The visualized network diagram of TFs, miRNAs, mRNAs, and their interactions was performed [19]. The enriched pathway analysis of the identified differentially expressed miRNAs was also performed with DIANA miRPath 3.0 [20].

### RT-qPCR

The differentially expressed miRNAs were further validated by RT-qPCR. The expression of selected miRNAs was performed using the Stem-loop TaqMan miRNA RT Kit (Invitrogen, Shanghai, China) and TaqMan RT assay according to the manufacturer protocols. A fixed volume of cDNA (1.2 μl) was combined with TaqMan universal PCR master mix II no UNG. The stem-loop sequences were UGCCCAGGCUGGAGCGAGUGCAGUGGUGCAGUCAGUCCUAGCUCACUGCAGCCUCGAACUCCUGGGCU for miR-3135b, GGGUAAGUGGAAAGAUGGUGGGCCGCAGAACAUGUGCUGAGUUCGUGCCAUAUGUCUGCUGACCAUCACCUUUAGAAGCCC for miR-654a-5p, and CCGAUGUGUAUCCUCAGCUUUGAGAACUGAAUUCCAUGGGUUGUGUCAGUGUCAGACCUCUGAAAUUCAGUUCUUCAGCUGGGAUAUCUCUGUCAUCGU for miR-146a-5p. The relative expression levels of target miRNAs were compared between samples using the comparative cycle threshold (Ct) method 2^(−ΔΔCt)^ after normalization to cel-miR-39 as an exogenous control.

### Statistical analyses

Statistical analysis was performed by SPSS Statistics for Windows software version 19.0 (IBM, Armonk, NY, USA) with student’s *t* test. We measured the area under the curve (AUC) of the receiver operating characteristic (ROC) curve for the validated miRNAs. The discrimination capability of the simple risk score was also presented by ROC curve. *p *< 0.05 was considered statistically significant.

## Results

### Clinical characteristics of the study population

No significant difference was observed in the age, estimated glomerular filtration rate (eGFR) [21], and creatine levels among active LNIV, active LNIV-CC, inactive LNIV, and healthy controls. Active LNIV group had higher Systemic Lupus Erythematosus Disease Activity Index (SLEDAI) scores and more 24-h proteinuria than inactive LNIV group (*p* < 0.01). Moreover, the 24-h proteinuria was lower in the active LNIV-CC group than the active LNIV group (*p* < 0.05) (Table 1).

**Table 1:** Baseline clinical data of the screening groups (mean ± SD)

Characteristic	Active LNIV-CC (n = 5)	Active LNIV (n = 5)	Inactive LNIV (n = 4)	Healthy controls (n = 3)
Age	24.6 ± 10.71	27.8 ± 12.50	28 ± 15.79	39.67 ± 10.66
Gender	Female	Female	Female	Female
eGFR (ml min^−1^ 1.73 m^−2^)	91.85 ± 14.77	96.20 ± 12.78	110.55 ± 5.69	121.3 ± 13.33
Proteinuria (mg day^−1^)	1819 ± 973.69^a,b^	4080.4 ± 1878.75^a^	220.33 ± 178.01	
SLEDAI	12.2 ± 1.79^a^	13.4 ± 4.22^a^	2 ± 1.63	
C3 (mg dl^−1^)	0.44 ± 0.10	0.37 ± 0.25	0.58 ± 0.37	
C4 (mg dl^−1^)	0.08 ± 0.03	0.07 ± 0.05	0.14 ± 0.10	
Cr (mg dl^−1^)	2.11 ± 0.74	1.02 ± 0.51	0.73 ± 0.23	0.60 ± 0.1

Cr, creatine; eGFR, estimated glomerular filtration rate; SLEDAI, Systemic Lupus Erythematosus Disease Activity Index

^a^*p *< 0.01 compared to inactive LNIV

^b^*p *< 0.05 compared to active LNIV without cellular crescents

### Characteristics of isolated urinary exosomes

**Fig. 1:**
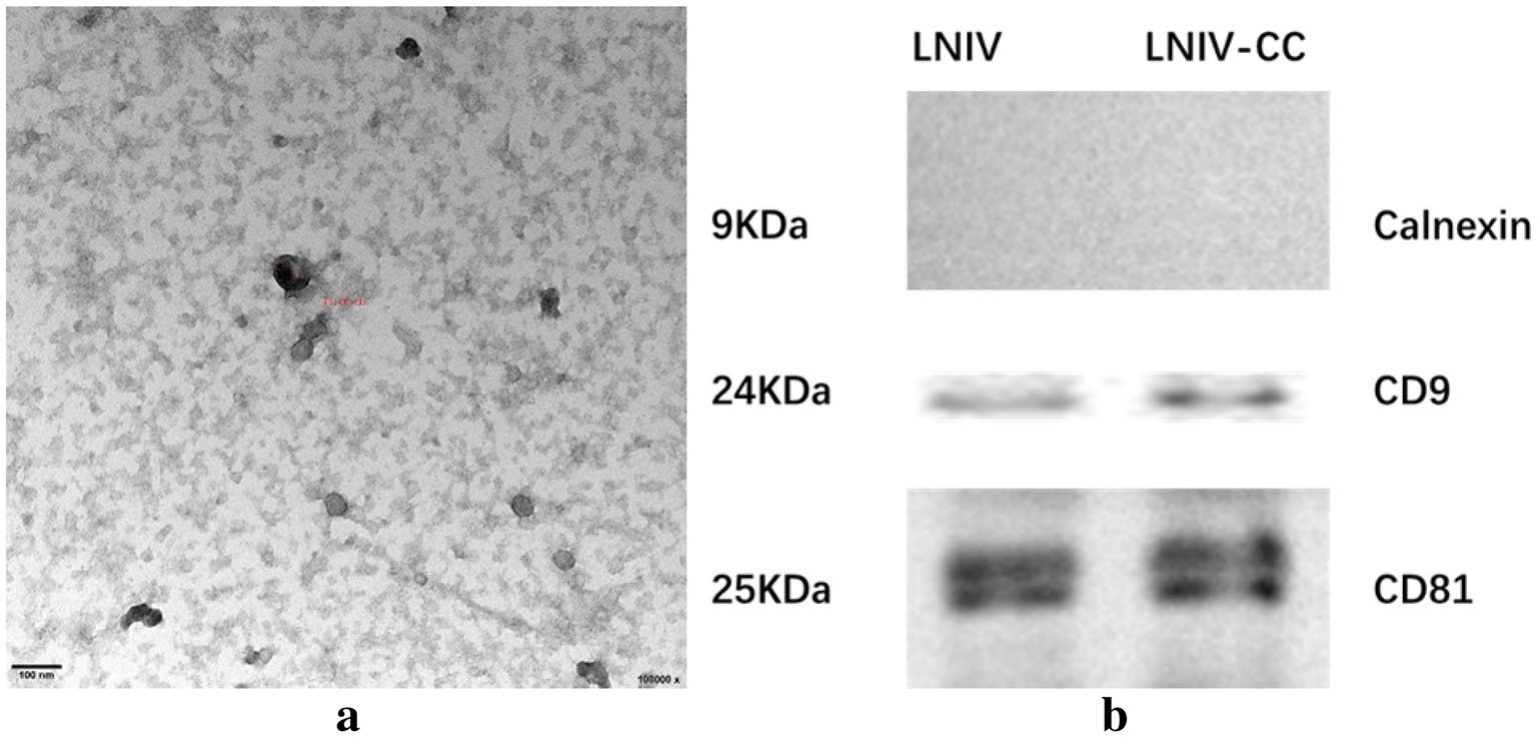
Characteristics of urinary exosomes. **a** Transmission electron microscope images of isolated exosomes. Bars = 100 nm. Diameter of the exosome measured at 74.66 nm. **b** Western blot indicated CD9 and CD81-positive expression and calnexin-negative expression

The extracted microvesicles were cup-shaped, < 100 nm in diameter, and scattered (Fig. 1a). Western blotting results showed positive expression of CD81 and CD9, and negative expression of calnexin (Fig. 1b), which is consistent with the typical characteristics of exosomes. Thus, the isolated microvesicles were exosomes.

### Differential expression of exosomal miRNAs

**Fig. 2:**
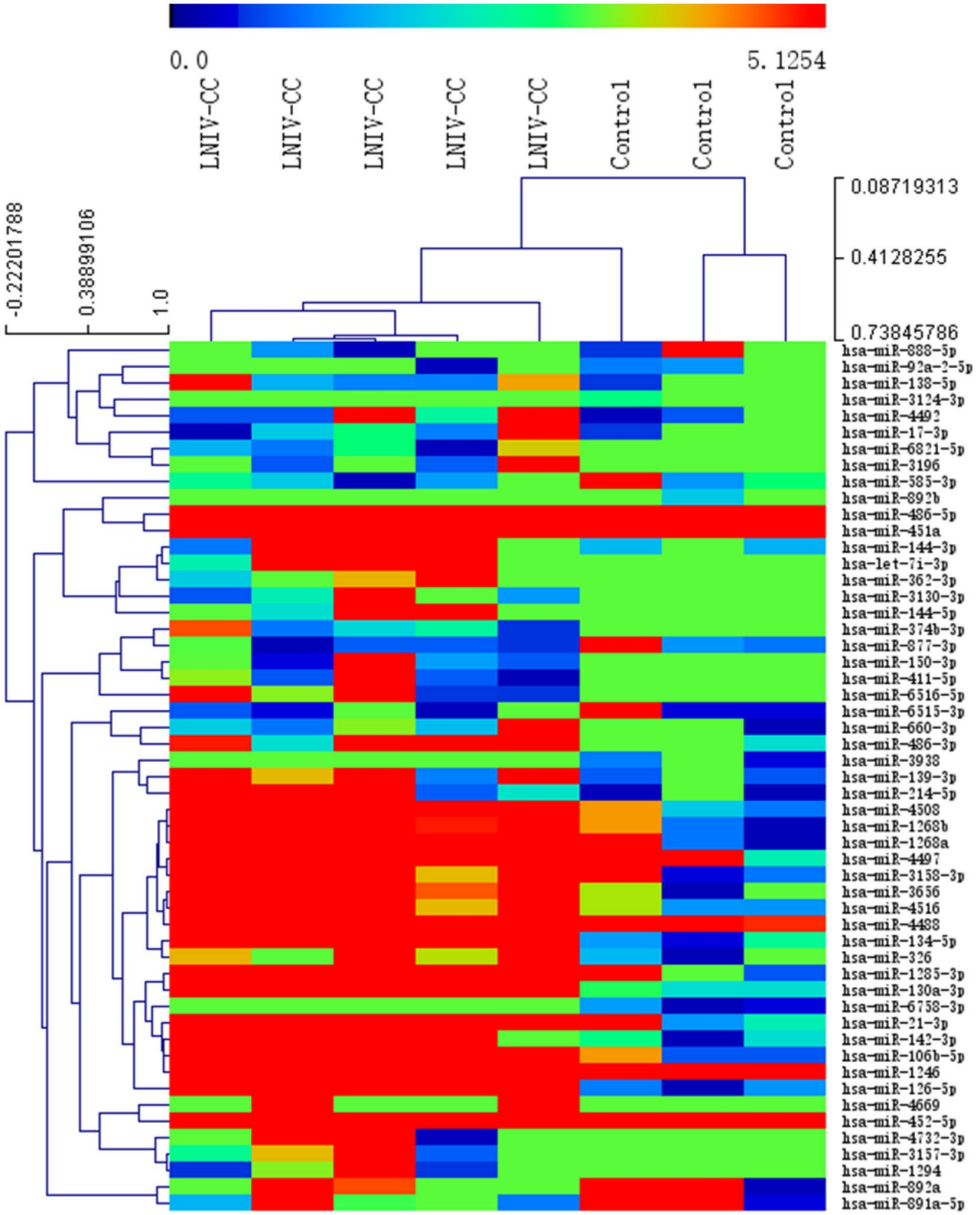
Heatmaps of differentially expressed miRNAs (*p* < 0.05) between LNIV-CC and control groups

**Fig. 3:**
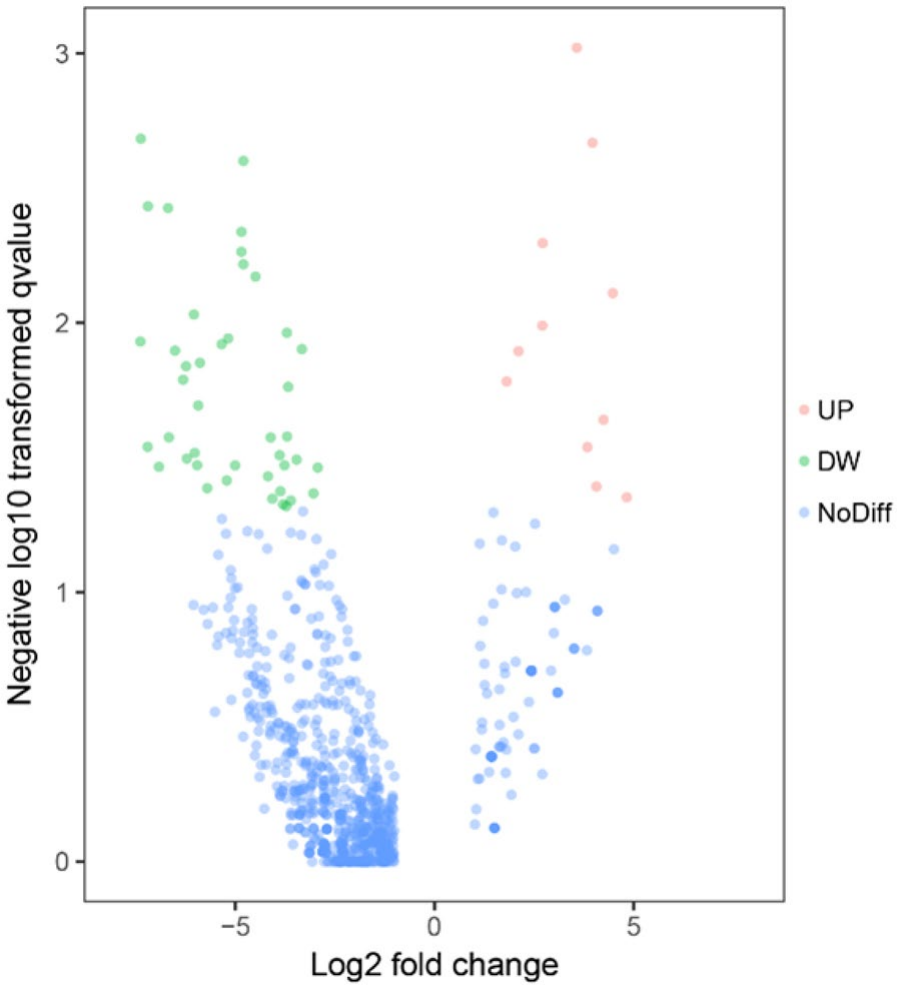
Volcano plots of differentially expressed miRNAs (*p* < 0.05) between LNIV-CC and control groups

**Fig. 4:**
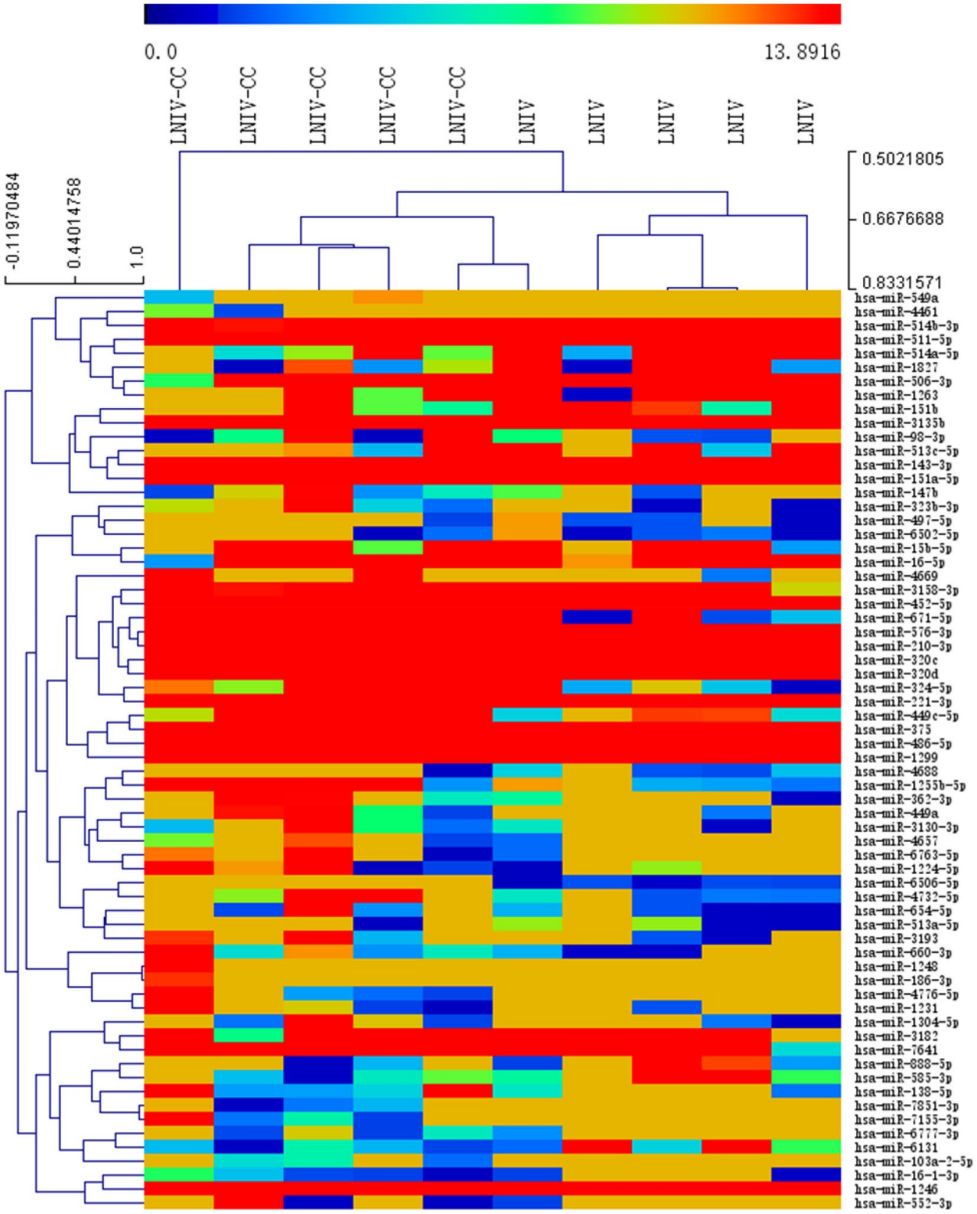
Heatmaps of differentially expressed miRNAs (*p* < 0.05) between LNIV-CC and LNIV (active) groups

**Fig. 5:**
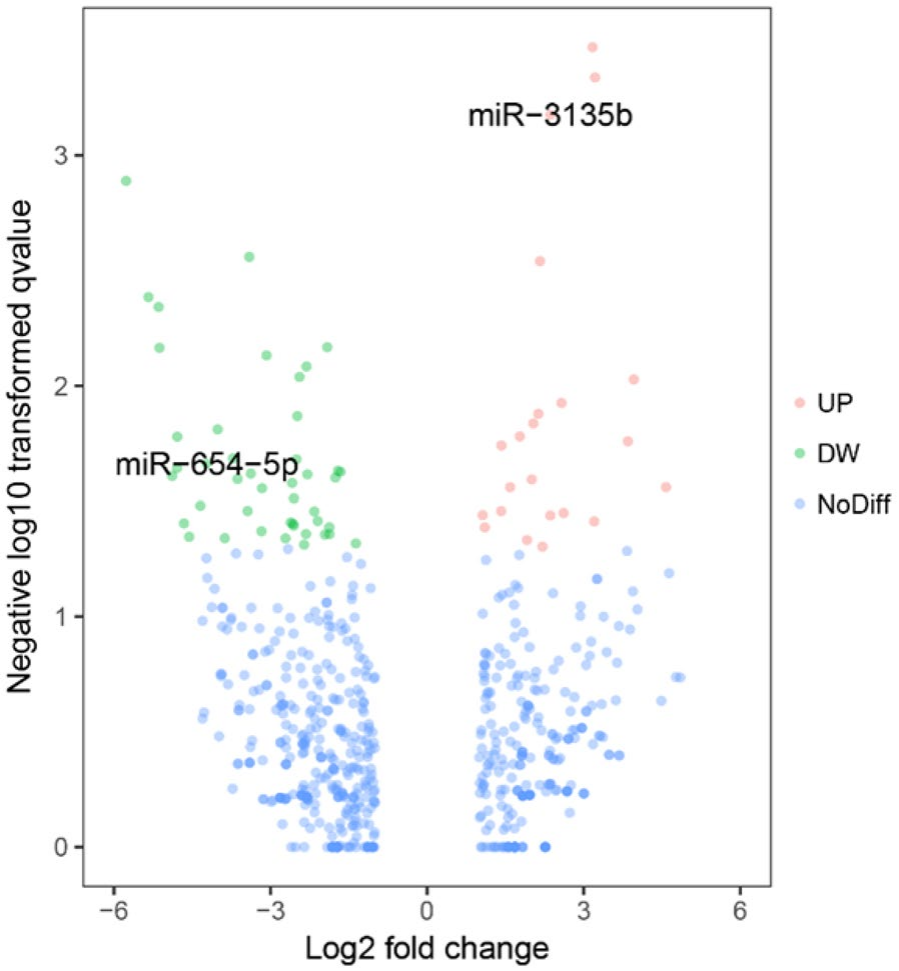
Volcano plots of differentially expressed miRNAs (*p* < 0.05) between LNIV-CC and LNIV (active) groups

A total of 904 altered exosomal miRNAs were detected between the LNIV-CC and control groups, including 53 with statistical significance (|log2 fold-change| > 1 and *p* < 0.05; Figs. 2 and 3). There were 662 common differentially expressed exosomal miRNAs detected between the LNIV-CC and LNIV (active and inactive) groups, including 66 with a significant difference (|log2 fold-change | > 1 and *p* < 0.05; Figs. 4 and 5). Among these differentially expressed miRNAs, a relative high expressed miR-3135b in LNIV (compared with other group), a relative high expressed miR-654-5p in LNIV-CC (compared with other group), and a not statistically significant differential expressed miR-146a-5p between LNIV-CC and LNIV (but higher than control group) were selected for validation with RT-qPCR.

### Validation of differentially expressed exosomal miRNAs by RT-qPCR

**Fig. 6:**
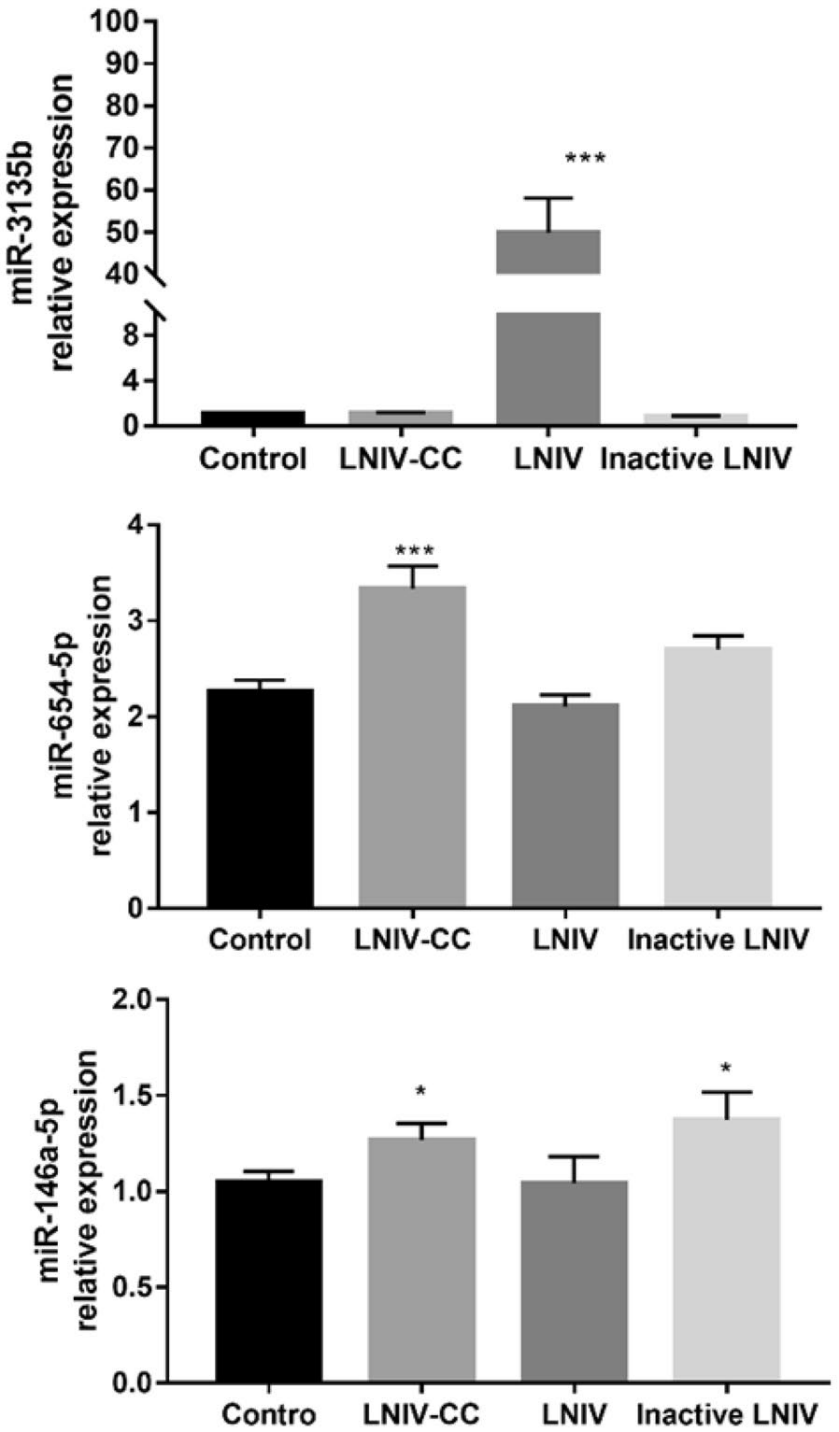
Expression of selected exosomal miRNAs for each group. Each value represents the mean ± SEM; *p < 0.05, ***p < 0.001 compared with control

For validation, RT-qPCR showed that the miR-3135b expression level in the active LNIV group was significantly higher than all other groups (*p* < 0.001), whereas the miR-654-5p expression level in the LNIV-CC group was significantly higher than LNIV and control groups (*p* < 0.001). The expression levels of miR-146a-5p in the inactive LNIV (*p* < 0.05) and LNIV-CC (*p* < 0.05) groups were significantly higher compared to that of the control group (Fig. 6). These results were consistent with the sequencing patterns, thereby confirming the accuracy and reliability of the sequencing data.

### Pathway analysis of the differentially expressed exosomal miRNAs

DIANA miRPath (V3.0) [20] was used for pathway analysis of differentially expressed miRNAs between the LNIV-CC and LNIV groups with three databases including TargetScan, Tarbase 7.0, and microT-CDS v5.0. After obtaining the intersection of the results, 15 pathways related to crescents in LNIV were identified (Table 2), including Wingless–Int (Wnt) signalling pathway, signalling pathways regulating pluripotency of stem cells, focal adhesion, Hippo signalling pathway, neurotrophin signalling pathway, axon guidance, Forkhead-box Class O (FoxO) signalling pathway, ubiquitin-mediated proteolysis, epidermal growth factor receptor (ErbB or EGFR) signalling pathway, TGF-beta signalling pathway, arrhythmogenic right, ventricular cardiomyopathy, lysine degradation, N-Glycan biosynthesis, other types of O-glycan biosynthesis, and fatty acid biosynthesis. The number of identified miRNAs in those pathways were also shown (Table 2). Wnt signalling pathway, pathways regulating pluripotency of stem cells, focal adhesion, Hippo signalling pathway, neurotrophin signalling, axon guidance, and FoxO signalling pathway were correlated with more than 50 identified differentially expressed miRNAs, respectively.

**Table 2:** Fifteen signalling pathways affected in LVIN-CC

KEGG pathway	*p* value	N genes	N miRNAs
Wnt signalling pathway	0.00020396	100	59
Signalling pathways regulating pluripotency of stem cells	0.001959962	95	57
Focal adhesion	0.002590298	138	56
Hippo signalling pathway	3.53E−05	107	54
Neurotrophin signalling pathway	0.009351432	81	54
Axon guidance	1.45E−05	94	52
FoxO signalling pathway	0.00541505	92	52
Ubiquitin-mediated proteolysis	0.006921462	91	49
ErbB signalling pathway	0.000164182	67	49
TGF-beta signalling pathway	0.004491223	53	48
Arrhythmogenic right ventricular cardiomyopathy	0.00042872	51	46
Lysine degradation	0.00109878	34	46
N-Glycan biosynthesis	0.009852971	34	39
Other types of O-glycan biosynthesis	0.034708487	20	32
Fatty acid biosynthesis	3.53E−05	8	11

These pathways were identified from the intersection of the DIANA-mirPath results of three databases (TargetScan, Tarbase7.0, and microT-CDS v5.0) with exclusion of tumour, hepatitis B, leukaemia, and thyroid hormone-related pathways

### TF–miRNA–mRNA network

**Fig. 7:**
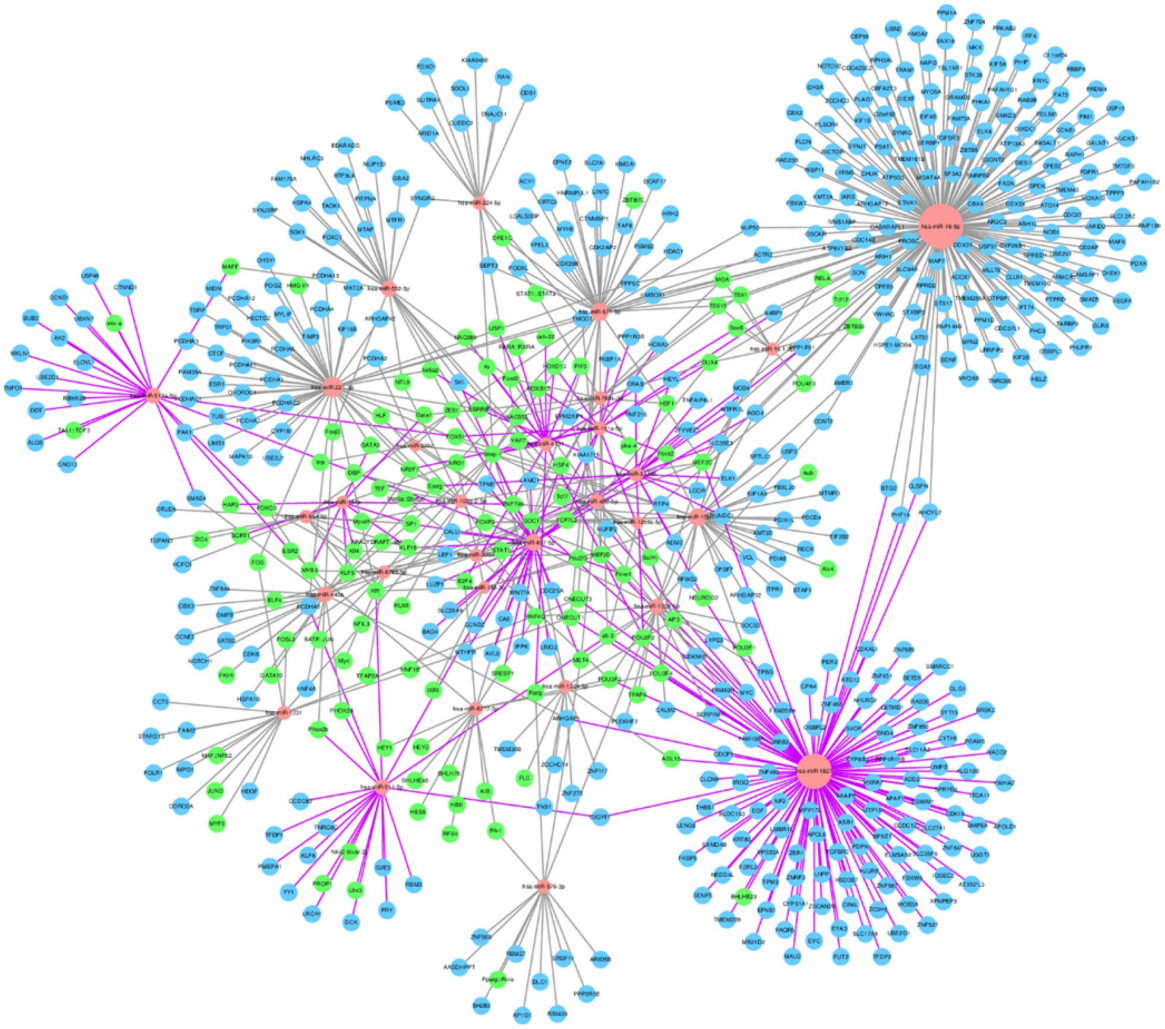
Network interactions between miRNAs, TFs, and target genes. The red circular nodes are miRNAs, the blue circular nodes are miRNA gene targets, and the green nodes are TFs. The grey line connects with down-regulated miRNAs, and the purple line connects with up-regulated miRNAs

The network diagram of TFs, miRNAs, mRNAs, and their interactions are shown in Fig. 7. Each miRNA node is connected to its TFs and targets with connecting lines. The more connections indicate a larger, more complicated and important miRNA node. Results indicated that the most important miRNAs included miR-1827, miR-16-5p, miR-671-5p, miR-324-5p, miR-552-3p, miR-22-5p, miR-513a-5p, miR-1231, miR-511-5p and miR-576-3p. This network diagram indicates important regulatory nodes in LNIV-CC.

### Prediction value of miR-3135b, miR-654-5p, and miR-146a-5p

**Fig. 8:**
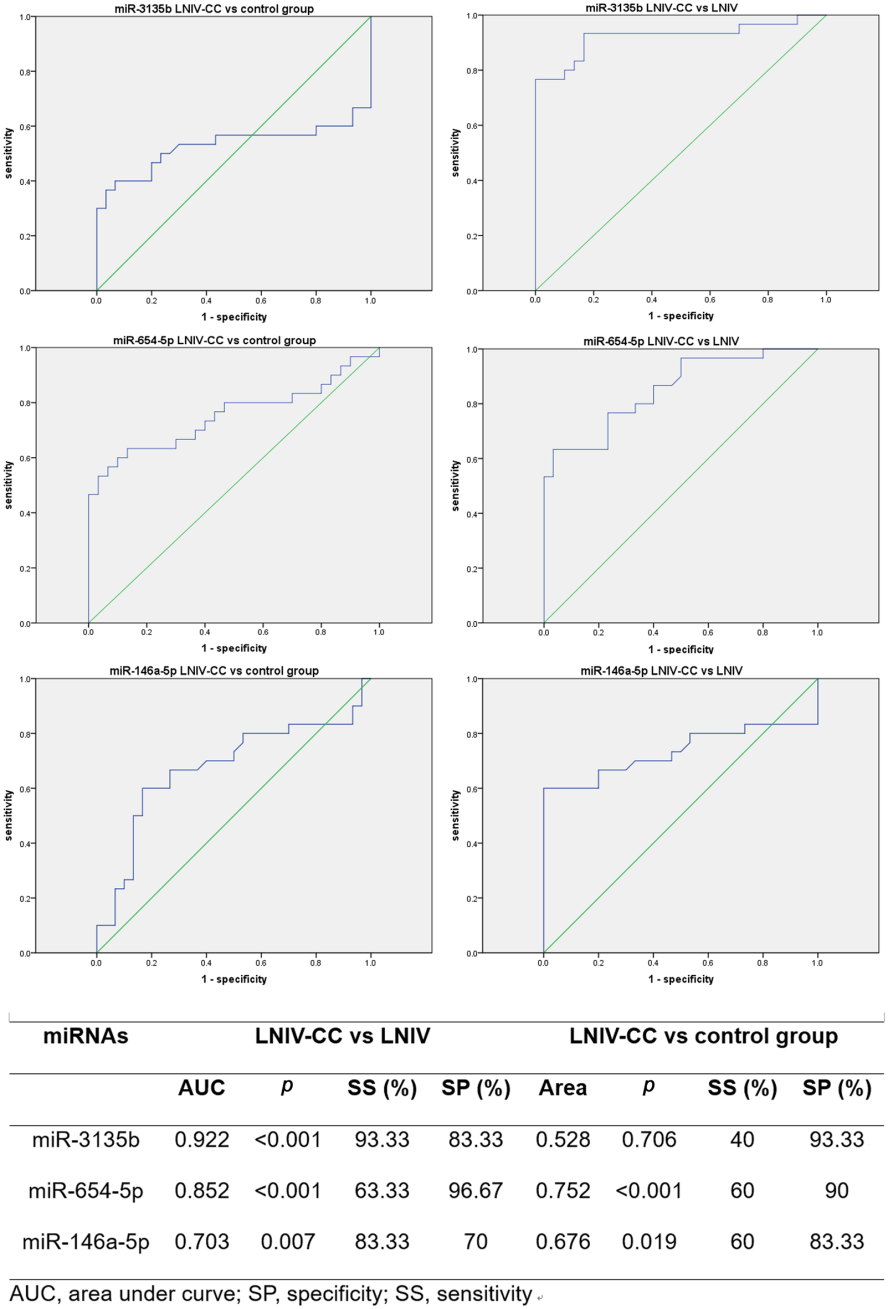
ROC curve for the validated miRNAs

To evaluate the prediction value of miR-3135b, miR-654-5p, and miR-146a-5p, receiver operating characteristic (ROC) analysis was performed (Fig. 8). In LNIV-CC vs. LNIV, the specificity and sensitivity of miR-3135b were 93.33% and 83.33%, those of miR-654-5p were 63.33% and 96.67%, and those of miR-146-5p were 83.33% and 70%, respectively. In LNIV-CC vs. control, the specificity and sensitivity of miR-3135b were 40% and 93.33%, those of miR-654-5p were 60% and 90%, and those of miR-146-5p were 60% and 83.33%, respectively. Thus, miR-3135b, miR-654-5p, and miR-146a-5p were of significantly prediction values for LNIV-CC.

## Discussion

miRNAs play an important role in biological function and renal pathology [12, 22]. Circulating miRNAs and urinary free miRNAs have been screened for diagnostic and therapeutic application in the renal pathology in lupus [23, 24]. However, those free miRNAs in body fluids are affected by enzymatic hydrolysis and therefore might not accurately reflect the pathological changes. Exosome is a new identified mode of intercellular communication. The miRNAs carried in exosomes can retain information on biological activity and quantity. Therefore, analysis of miRNAs in urinary exosomes might be more valuable. This study was the first to analyse the characteristics of miRNA expression in exosomes from LNIV-CC patients.

We demonstrated that the miRNA expression profile of urinary exosome in LNIV-CC is distinct from other groups, and miR-3135b and miR-654-5p are candidate biomarkers of LNIV-CC. The specificity of prediction of them was as high as 83.33–96.67%, which indicates the value of these miRNAs for further research and potential clinical application. miR-146a expression levels were also higher in the LNIV-CC group than the control group. miR-146a has been reported as a marker of glomerular and tubular interstitial tissue injury [25, 26]. The miR-146a expression level in lupus nephritis patients is not consistent. It was previously reported to be significantly higher in lupus nephritis patients than control group [27] but other studies showed that miR-146a is down-regulated in renal tissues and in mononuclear cells of patients with lupus nephritis compared to controls [28, 29]. Here, we did not detect a significant difference between the LNIV-CC and LNIV groups. It was suggested that miR-146a-5p likely does not play a major role in the regulation of CC that is characterized by substantial epithelial cell proliferation and monocyte infiltration.

miR-3135b is a biomarker of coronary artery calcification, heart failure, and non-ST segment elevation acute coronary syndromes, which is closely related to a poor cardiovascular prognosis [30, 31]. Thus, elevation of miR-3135b in the LNIV without crescents group may suggest that those patients are more likely to develop systemic lupus erythematosus-related cardiac damage. Our study firstly reports miR-3135b in association with kidney disease.

miR-654-5p has been reported to promote carcinoma proliferation in oral squamous cell through the miR-654-5p/GRAP/Ras/Erk signalling pathway [32], and down-regulation of miR-654-5p inhibited the proliferation of prostate cancer cells [33]. Thus, the high expression of miR-654-5p is closely related to the abnormal cell proliferation. CCs are formed because of the abnormal proliferation and more than three layers of epithelial cells. Since miR-654-5p was found to be highly expressed in the LNIV-CC group, it may be involved in the abnormal proliferation of epithelial cells.

There are several limitations in the present study. First, the number of subjects was relatively small. We only performed miRNA sequencing on only 17 patients, and validated the data on additional 40 patients. Therefore, large-scale cohort studies are needed to further analyse and validate these results, especially for diagnosis. Second, this is a cross-sectional study, and thus follow-up after treatment of lupus nephritis is needed to confirm the findings. Third, recent reports suggest that exosomes may cross the blood–brain barrier through receptor-mediated transcellular transport [34, 35]; however, it is currently not known whether this mechanism plays a role in the renal filtration barrier. Finally, the present study lacks functional experiments at cellular or molecular level to verify the causal relationship between miRNAs and lupus nephritis. We described the enriched signalling pathways with number of identified miRNAs in this study but the functional work regarding the miRNAs identified and the signalling pathways enriched should be performed in the future.

Overall, we demonstrate that LNIV with crescents has a unique miRNA expression profile of urinary exosome and complex regulatory network, and found that miR-3135b, miR-654-5p and miR-146a-5p in urinary exosomes could be used as novel non-invasive diagnostic markers for LNIV with crescents. Moreover, we identified 15 signalling pathways potentially associated with the formation of crescents in LNIV and established a visualized network of TF–miRNA–mRNA interactions, which lays the foundation for further studies on the pathology of lupus nephritis.

## Abbreviations

LNIV: Type IV lupus nephritis
LNIV-CC: Type IV lupus nephritiscomplicated with cellular crescent
miRNA: microRNA
TFs: transcription factors
RT-qPCR: reverse transcription-quantitative polymerase chain reaction
KDIGO: “the Kidney Disease: Improving Global Outcomes”
eGFR: estimated glomerular filtration rate
SLEDAI: Systemic Lupus Erythematosus Disease Activity Index
Cr: creatine
Wnt: Wingless–Int
FoxO: Forkhead-box Class O
ErbB or EGFR: epidermal growth factor receptor
